# Food assistance is associated with decreased nursing home admissions for Maryland’s dually eligible older adults

**DOI:** 10.1186/s12877-017-0553-x

**Published:** 2017-07-24

**Authors:** Sarah L. Szanton, Laura J. Samuel, Rachel Cahill, Ginger Zielinskie, Jennifer L. Wolff, Roland J. Thorpe, Charles Betley

**Affiliations:** 1 Johns Hopkins University School of Nursing, 525 N Wolfe street, Baltimore, Maryland 21205 USA; 2 Johns Hopkins University Bloomberg School of Public Health, Baltimore, Maryland USA; 3 Benefits Data Trust, Philadelphia, PA USA; 4 Hilltop Institute, University of Maryland, Baltimore, USA

## Abstract

**Background:**

Although it has long been known that a broad range of factors beyond medical diagnoses affect health and health services use, it has been unclear whether additional income can decrease health service use. We examined whether Supplemental Nutrition Assistance Program (SNAP) receipt is associated with subsequent nursing home entry among low income older adults.

**Methods:**

We examined the 77,678 older adults dually eligible for Medicaid and Medicare in Maryland, 2010–2012. Zero inflated negative binomial regression, adjusting for demographic and health factors, tested the association of either lagged SNAP enrollment or lagged benefit amount with nursing home admission. We used Heckman two-step model results to calculate potential savings of SNAP enrollment through reduced nursing home admissions and reduced duration.

**Results:**

Only 53.4% received SNAP in 2012, despite being income-eligible. SNAP participants had a 23% reduced odds of nursing home admission than nonparticipants (95% CI: 0.75–0.78). For SNAP participants, an additional $10 of monthly SNAP assistance was associated with lower odds of admission (OR = 0.93, 95% CI: 0.93–0.93), and fewer days stay among those admitted (IRR = 0.99, 95% CI: 0.98–0.99). Providing SNAP to all 2012 sample nonparticipants could be associated with $34 million in cost savings in Maryland.

**Conclusions:**

SNAP is underutilized and may reduce costly nursing home use among high-risk older adults. This study has policy implications at the State and Federal levels which include expanding access to SNAP and enhancing SNAP amounts.

**Electronic supplementary material:**

The online version of this article (doi:10.1186/s12877-017-0553-x) contains supplementary material, which is available to authorized users.

## Background

It has long been established that a broad range of factors beyond medical diagnoses affect health and health services use [1]. An example is food insecurity, which is experienced by 4.8 million older adults [2], and is linked to poor mental and physical health status [3]. Older adults who experience food insecurity report limitations in activities of daily living 14 years earlier than older adults who do not experience food insecurity [4]. Older adults who experience food insecurity are more likely to have hyperlipidemia, diabetes, heart disease, and high blood pressure than those older adults who do not experience food insecurity [5–7]. Among people with chronic illnesses, food insecurity is associated with cost-related medication underuse [8]. Among those with diabetes, food insecurity is associated with worse glycemic control and LDL control [9] and, for low income diabetics, increased hospital admissions at the end of the month when funds can run low [10]. As another example, older adults who report financial strain (not enough money to pay their bills) are more likely to suffer depression, disability, malnutrition and early mortality [11–13] compared to those with similar incomes.

Although we know that food insecurity and financial strain are related to poor health outcomes and increased health care costs [14], little is known about whether receipt of Supplemental Nutrition Assistance Program (SNAP) - a federal entitlement that ameliorates food insecurity (Mabli, 2013) and may reduce financial strain – improves health outcomes and reduces health care costs. With the changes to the U.S. health care system that emphasize covered *lives* instead of covered *procedures*, it is particularly timely to ask whether social programs such as SNAP exert an effect on health services outcomes. This study specifically examines whether participation in a food assistance program, which reduces food insecurity and increases household income, is associated with a decreased risk for nursing home utilization.

SNAP is a United States Department of Agriculture program administered by States extended to individuals whose annual income is less than 130% of the Federal Poverty Level. In FY 2014, SNAP served 46 million people in an average month at an annual cost of $70 billion. A total of 9% of SNAP costs are directed to older adults [15]. SNAP benefits are distributed directly to enrolled participants on a debit card that can only be spent on food. Although SNAP is a federal entitlement program, states have some flexibility in establishing eligibility criteria, within certain parameters [16]. For example, using a mechanism known as categorical eligibility, Maryland (and several other states) have raised this income limit to 200% of the FPL. In Maryland, adults age 65 and older who are citizens or qualified immigrants and have income below 200% of the Federal Poverty Level are eligible for at least the minimum monthly SNAP benefit of $16 [17]. Participants may qualify for a higher SNAP benefit level by documenting deductible expenses, like rent, heat, and medical costs [17]. The average monthly benefit among older adults who receive SNAP in Maryland is $108, or $1248 per year [15]. The average gross income for SNAP households with older adults is only $883 per month. Based on that, the average SNAP benefit ($108) represents 12% of their income [18].

## Methods

### Study population

We studied Maryland residents ages 65 and older who were enrolled in both fee-for-service Medicare and Medicaid between 2009 and 2012. Because Medicaid eligibility is much more restrictive than the 200% poverty threshold for SNAP, those eligible for Medicaid are eligible for SNAP. Study eligibility was determined separately in each calendar year. We defined Medicaid enrollment on the basis of being enrolled in the program for one or more months during a calendar year and Medicare enrollment as being enrolled in Medicare for 6 or more months during a calendar year, without any Medicare Advantage enrollment. This mirrors the approach taken by a Department of Health and Human Services report studying housing and health [19]. To be included, individuals must have had at least 6 months Medicare eligibility. For people who turned 65 during the year, we included them in the sample if they were 65 for at least 6 months of the calendar year. We also included people who died during the sample year if they were alive for at least 6 of the 12 study months. Individuals who were continuously living in a nursing home for 9 months or more during the prior calendar year were excluded from the sample because nursing home residents are ineligible to receive SNAP benefits [20]. Overall, of 212,667 person-year observations during 2010–2012, 35,387 were excluded because of Medicare Advantage enrollment, 8418 were excluded because of death, and 19,376 were excluded because of Nursing Home stays in the previous year exceeding 270 days, leaving 149,486 observations in the sample.

### Data

We merged Medicaid claims and sociodemographic data with Medicare claims data and SNAP program utilization data from Maryland Human Resources data base for all individuals who met study eligibility criteria. Matching was performed on individual Social Security numbers available in the data sets provided by the Maryland state agencies. This was the most efficient and accurate match, since other identifying fields (e.g. address) were less reliably maintained and not always common to both the Medicaid, Medicare and SNAP data sets.

Each calendar year between 2010 and 2012 represents an individual observation period. Therefore, each participant was either a “yes” or “no” for nursing home admission each year, and each participant’s nursing home costs were calculated separately for each year. SNAP exposure was modeled with one year time lags [21].

### Nursing home utilization and costs

The dependent variables in this study relate to nursing home utilization. Nursing home utilization includes both post-acute skilled nursing facilities if they extended past the 90 day Medicare benefit limit as well as direct admission to a Medicaid nursing home for someone without a prior hospitalization. Nursing home utilization variables include annual count of nursing home admission days (including both short and long stays) and annual nursing home cost.

### SNAP enrollment and spending

The main independent variables were SNAP enrollment and SNAP benefit amount, modeled separately. SNAP enrollment is a binary measure. SNAP benefit was measured as the cumulative average monthly SNAP benefit received through the prior year, in $10 increments, to aid interpretation. The sample was restricted to participants who receive SNAP in the models for which SNAP benefit amount was the main independent variable.

### Covariates

Other variables included age, gender, race/ethnicity [analyzed as black, Caucasian, Hispanic, other and unknown because the Medicaid Management Information System reports with these categories], and annual household income (in $1000 increments). The Medicaid Management System does not allow separate adjustment for ethnicity and race. Ten percent were coded as “unknown” and were included in the analysis. We measured number of chronic medical conditions with a modified version of the Chronic Conditions Warehouse algorithm using diagnosis codes listed in both Medicare and Medicaid claims [22]. This is the standard method that Medicare uses to assess chronic conditions from claims data and is used to control for health status and not risk adjustment [22]. Enrollment in a Medicaid community based care waiver program was included because to enroll in such programs, older adults must meet a nursing home level of care based on impaired function, which means they are at higher risk for nursing home admission than the general population. We adjusted for the category of Medicaid enrollment (i.e. medically needy spend-down and partial eligibility) since the type of enrollment is a proxy for socioeconomic status [23]. We also adjusted for any nursing home use in the previous year to account for autocorrelation, and the year of observation to account for general trends over time. This study was approved by both the Johns Hopkins and the University of Maryland Baltimore County Institutional Review Board. Written human subject consent was deemed unnecessary by both institutional review boards because the data were unidentified and already collected. The data are not publicly available and reside only at the University of Maryland Baltimore County as the repository of state data.

### Statistical analysis

We first analyzed whether SNAP was related to nursing home admission and length of stay using zero inflated negative binomial regression models [24, 25]. Zero inflated negative binomial regression models fit the data due to the many non-nursing home users during the year and skewed distribution of nursing home days. We used robust standard error estimates to account for possible heteroscedasticity in the data. The cost models are similar. We used a Heckman two-step selection model [26] for the continuous spending variable [25] for the same reason as the zero-inflated negative binomial: there is more zero utilization in any given year than utilization. The Heckman model first estimates the probability of having any nursing home costs, and then uses a weighted ordinary least squares calculation to estimate the association between SNAP spending and nursing home cost, adjusted for the initial probability of having nursing home costs [26]. We adjusted all models for sex and race and time-varying values for age, annual income, chronic conditions, and enrollment in a Medicaid community waiver program, category of Medicaid enrollment, study year, autoregressive effects, and the cumulative monthly average lagged SNAP amount.

To understand the possible cost implications, we used the following multi-step process. First, we took results from the Heckman two-step model so that we could adjust for sociodemographic differences between SNAP and non-SNAP participants. We computed the marginal effect of receiving SNAP at mean values of all covariates in the first stage. This provides the difference in probability of admission associated with SNAP after adjusting for sociodemographic differences. We then multiplied this probability with the total number of non-SNAP participants and the average annual cost of nursing home stay to compute total cost savings from averted nursing home admission for non-SNAP participants. Next, we used the Heckman second stage to calculate possible cost savings through reduced duration for those SNAP participants who were admitted into a nursing home. This gives the estimated percentage change in cost associated with SNAP receipt for participants who enter a nursing home. The potential total cost savings from reduced nursing home use is the sum of the results of each stage.

## Results

Study participants were predominantly female (69%) and they were racially and ethnically diverse: 39% were Caucasian, 33% were Black, and 5% were Hispanic with 22% of unknown race/ethnicity as seen in Table 1. Participants were on average 76 years old and had 2.8 chronic conditions. Annual income of study participants was $5864 (data not shown). Although nearly all study participants would qualify for SNAP by virtue of their low incomes, only 38.6% received this benefit in years 2010–2012 (see Additional file 1: Table S1 for further information on the cohorts over time).

**Table 1:** Characteristics of the dually eligible older adults in the population studied

	2012 (*n* = 53,646)						
Variable	Receiving SNAP^c^		Not Receiving SNAP^c^		Total		*p* value
	(*n* = 28,628)	(53.4%)	(*n* = 25,018)	(46.6%)			
Age							*p* < 0.001
65–69	7305	29%	7367	26%	14,672	27%	
70–74	4406	18%	7215	25%	11,621	22%	
75–79	4164	17%	5812	20%	9976	19%	
80–84	3693	15%	4405	15%	8098	15%	
≥ 85	5450	22%	3829	13%	9279	17%	
Gender							*p* = 0.0105
Female	19,955	70%	17,183	69%	37,138	69%	
Male	8673	30%	7835	31%	16,508	31%	
Race/Ethnicity							*p* < 0.001
Black	10,191	36%	7513	30%	17,704	33%	
Caucasian	10,560	37%	10,474	42%	21,034	39%	
Hispanic	1694	6%	1175	5%	2869	5%	
Other	4281	15%	2554	10%	6835	13%	
Unknown	1902	7%	3302	13%	5204	10%	
HCBS^a^ Waiver Status							*p* = 0.361
No	24,903	87%	21,829	87%	46,732	87%	
Yes	3725	13%	3189	13%	6914	13%	
Has QMB/SLMB^b^							*p* < 0.001
No	16,984	59%	14,363	57%	31,347	58%	
Yes	11,644	41%	10,655	43%	22,299	42%	
Received Medicaid through Spenddown							*p* < 0.001
No	28,207	99%	24,516	98%	52,723	98%	
Yes	421	1%	502	2%	923	2%	
Mean Number of Chronic Conditions	2.6		2.9		2.8		*p* < 0.001
Admitted to nursing facility							*p* < 0.001
No	26,486	93%	19,808	79%	46,294	86%	
Yes	2142	7%	5210	21%	7352	14%	

^a^Home & Community Based Services

^b^QMB – Qualified Medicare Beneficiary; SLMB – Specified Low-Income Medicare Beneficiary

^c^SNAP – Supplemental Nutrition Assistance Program

Approximately 14% of study participants were admitted to a nursing home in 2012 as shown in Table 1 with 17% being admitted to a nursing home at any time in the 3 years. Participants in SNAP had a 23% lower odds of subsequent nursing home admission than their counterparts who did not receive SNAP (aOR = 0.77, 95% CI 0.75, 0.78; Table 2). An additional $10 of SNAP assistance per month for those receiving SNAP was associated with lower odds of nursing home admission (OR = 0.93, 95% CI: 0.93,.93), and for those admitted, incidence rates of additional days were lower (IRR = 0.99, 95% CI: 0.98–0.99 for SNAP) as shown in Table 2. Using the cost data, we found that receiving SNAP was associated with reduced nursing home admissions of 2.67 percentage points (95% CI: -2.3,-3.0), and for those admitted, receiving SNAP was associated with a reduction in nursing home cost of 12% (95% CI: -8,-15). An additional $10 of SNAP assistance per month for those receiving SNAP was associated with lower admission rate of .44 percentage points (95% CI −.42,-.47), and for those admitted, each additional $10 of SNAP assistance per month for those receiving SNAP was associated with lower cost of 4% (95% CI: 3,6). Among those admitted to a nursing home, in our study sample, the average annual nursing home cost was $28,360 including short term (Medicare-funded) and longer term (Medicaid-funded) nursing home care.

**Table 2:** Odds of nursing home utilization by SNAP participation

Nursing Facility Utilization		
	Admissions^a^	Expenditures^b^
*Predictor Variables*	*Likelihood of nursing facility use*	*Any nursing facility use*
	Odds Ratio (95% CI)	Marginal Effect for any >$0 nursing facility cost evaluated at means
Any SNAP	0.77 (0.75, 0.78)	−2.67% (−2.3%, −3.0%)
For those >$0 SNAP,cumulative monthly mean SNAP amount ($10)	0.928 (0.926, 0.931)	−0.44% (−0.42%, −0.47%)
	Number of nursing facility days among those using facilities	Nursing facility cost among those using facilities
	Incident Rate Ratio for SNAP Use (95% CI)	% Change in cost for SNAP use (95% CI)
Any SNAP	0.92 (0.89, 0.95)	−12% (−8%, −15%)
	Incident Rate Ratio for $10 Change in SNAP (95% CI)	% Change for cost for $10 change in SNAP (95% CI)
For those >$0 SNAP, cumulative monthly mean SNAP amount ($10)	0.99 (0.98, 0.99)	−4% (−3%, −6%)

All models adjusted for autoregressive effects, age, sex, race/ethnicity, annual income, chronic condition count, enrollment in the Medicaid community waiver program, and Medicaid partial dual eligibility or spend-down eligibility

^a^Associations estimated from zero-inflated negative binomial regression estimated with robust standard errors

^b^Associations estimated from Heckman regression model

Based on the estimated effect of receiving SNAP, an additional 667 of the 25,018 non-SNAP participants in 2012 could have avoided going to a nursing home, giving total possible savings from averted nursing home admissions of $19 million. After taking into account this reduction in nursing home admissions, 4543 non-SNAP participants in 2012 would still have been admitted to a nursing home. For these participants, based on the estimated effect of receiving SNAP in the second stage of the Heckman model (12%), SNAP receipt could have reduced cost by $15 million. Thus, giving SNAP to the 25,018 non-SNAP participants in the sample could have been associated with savings of $34 million in 2012.

## Discussion

To our knowledge, this is the first study to examine the relationship between SNAP benefits and nursing home admissions among low-income older adults. Study findings align with recent research showing that home delivered meals are associated with lower rates of nursing home admissions [27, 28]. In Maryland’s dually eligible older adults, only about half received food assistance benefits and of those who received benefits, the average benefit was $117 per month, while 15% received the minimum benefit of $16 per month. Our findings that higher SNAP benefits are associated with fewer nursing home admissions suggest that greater spending on food assistance may reduce nursing home utilization.

### Limitations to the methods

Limitations of this study include the following. First, we do not have access to information about and are unable to assess lifetime exposure to SNAP, food insecurity, lifetime access to health insurance, or housing adequacy. Second, our comprehensive approach to measurement and the inclusion of income and chronic conditions in our statistical models will lead to conservative estimates of association between food assistance and nursing home admission. Third, some individuals may have benefitted from food assistance programs other than SNAP such as food banks or Meals on Wheels, but information about other food assistance programs was not available to us. This could bias the results in a conservative direction. Fourth, people who enroll in SNAP may be fundamentally different in ways we have been unable to measure than people who do not, such as the ability to navigate the complex enrollment process. Due to this selection bias, the literature on SNAP has been somewhat mixed. While it is clear that SNAP decreases food insecurity [29], and minimizes the adverse effect of food insecurity on dietary quality and obesity [30]. some studies have found greater obesity rates in women who participate in SNAP than in eligible nonparticipants (as summarized by a recent review by DeBono and colleagues) [31]. However, those studies may have been confounded by poor access to healthy foods in low income communities [32] and biased by self-selection into the SNAP program; this association is not consistently found in longitudinal studies that apply instrumental variable or difference in difference techniques to account for self-selection [33–36]. Because random assignment is not feasible for SNAP inferences regarding non-SNAP participants must be done with caution and we cannot assert causality. Due to the second stage modeling however, we can conclude that higher SNAP benefits among program participants are associated with a protective effect, since we are comparing SNAP participants to each other.

### Strengths of the methods

Unlike many studies that analyze income by self-report with resulting missing data, each person in this data set provided income documentation to enroll for Medicaid. All of our statistical models controlled for income so these findings show that SNAP is associated with decreased nursing home placement while adjusting for income. This study is strengthened by time lagged measurement of SNAP spending, which ensures that receipt of SNAP benefits temporally precedes nursing home use. Another strength of the study is that we examine the entire population of interest in one state with objective measurement of SNAP receipt rather than self-report. A final strength is the ability to look at “dose” of SNAP in $10 increment. In our analyses, those with higher benefits (but same income) are less likely to be admitted to a nursing home and/or have shorter nursing home stays. Since prior work has focused on the SNAP enrollment effect, our results contribute to the literature by demonstrating a dose-response association between benefit amounts and nursing home stays.

The possible savings due to averted nursing home admissions found in this study is striking. Findings suggest States could reap Medicaid savings if they could invest in efforts to enroll more older adults in SNAP. Based on expenditure accounts, $148 billion was spent nationally on nursing care facilities and continuing care retirement communities in 2012 [37]. If weighted by state population, Maryland would have spent $2.8 billion. The cost estimate of $34 million dollars in 2012 (based on the second method) is then 1.2% of this spending, a non-negligible amount. These savings estimates do not include the cost of providing SNAP which is a Federal expense. Based on the average per capita costs of the SNAP program, we estimate that the federal government would spend approximately $39 million if it extended SNAP benefits to the 2012 income eligible nonparticipants in our sample. Ignoring inefficiencies in taxation, the $34 million would be almost paid for in savings in health care utilization of nursing homes alone. This does not count additional health savings on the pathway to nursing homes such as hospitalization or increased primary and specialty care. Also, States do not pay for SNAP but they do pay for their share of Medicaid. So, states may obtain net savings by increasing SNAP participation and benefit amounts.

## Conclusions

Increasing food access for low income older adults may reflect a cost efficient way of reducing utilization while improving quality of life which is a top priority of CMS [38].

### Public health implications

#### Policy implications

This study has policy implications at both State and Federal levels. Our findings are consistent with research showing that older adults residing in States that spend a higher proportion of money on social services compared to medical care have better daily functioning and health outcomes in the year after this spending [39]. Seven States (Alabama, South Carolina, Florida, Georgia, Mississippi, Washington, and Pennsylvania) are currently testing new models that decrease SNAP enrollment barriers by extending certification periods from 12 to 36 months, waiving re-certification interviews, or leveraging electronic data to reduce verification requirements [40]. Other states, including Pennsylvania, are leveraging administrative data to identify individuals who are not receiving benefits for which they are entitled [41]. Another possible State action is to increase the minimum monthly SNAP benefit. The State of Maryland recently passed legislation to increase the minimum monthly SNAP benefit from $16 to $30 using State dollars [42]. Public and private health care payers could screen beneficiaries for food security and financial strain as a way to systematically identify and connect low-income individuals to food assistance [43]. On the Federal level, Social Security could leverage its information about low-income seniors to streamline enrollment into SNAP.

The National Association of State Budget Officers estimates that in Fiscal year 2015 Medicaid comprised 27.4% of total State expenditures [44]. State spending on Medicaid is expected to rise as the age of the population increases and more older adults rely on Medicaid to pay for nursing home use. Identifying policy levers to reduce nursing home costs through averting avoidable utilization is an important public health goal.

## Additional file

Additional file 1: Table S1. Characteristics of study population across three year study period. (DOCX 17 kb)
