# Supplementary material for: Food assistance is associated with decreased nursing home admissions for Maryland’s dually eligible older adults
Source: BMC Geriatr. 2017 Jul 24;17:162. doi: 10.1186/s12877-017-0553-x (PMC5525341; doi:10.1186/s12877-017-0553-x)
Supplement: Additional file 1: Table S1. — Characteristics of study population across three year study period. (DOCX 17 kb) [file 12877_2017_553_MOESM1_ESM.docx]

|  | 2010-2012 (n= 149,184) | | | | |
| --- | --- | --- | --- | --- | --- |
| Variable | Receiving SNAP^3^ | | Not Receiving SNAP^3^ | | p value |
|  | (n=57,569) | (38.6%) | (n=91,615) | (61.4%) |  |
| Age |  |  |  |  | p<0.001 |
| 65-69 | 10,821 | 19 | 28,554 | 31 |  |
| 70-74 | 15,697 | 27 | 17,045 | 19 |  |
| 75-79 | 12,793 | 22 | 15,329 | 17 |  |
| 80-84 | 9,789 | 17 | 13,214 | 14 |  |
| ≥85 | 8,469 | 15 | 17,473 | 19 |  |
| Gender |  |  |  |  | p<0.001 |
| Female | 40,371 | 70 | 63,350 | 69 |  |
| Male | 17,198 | 30 | 28,265 | 31 |  |
| Race/Ethnicity |  |  |  |  | p<0.001 |
| Black | 19,344 | 34 | 30,000 | 33 |  |
| Caucasian | 22,359 | 39 | 37,184 | 41 |  |
| Hispanic | 3,327 | 6 | 4,443 | 5 |  |
| Other | 9,500 | 17 | 9,568 | 10 |  |
| Unknown | 3,039 | 5 | 10,420 | 11 |  |
| HCBS^1^ Waiver Status |  |  |  |  | p<0.001 |
| No | 49,264 | 86 | 82,819 | 90 |  |
| Yes | 8,305 | 14 | 8,796 | 10 |  |
| Has QMB/SLMB^2^ |  |  |  |  | p<0.001 |
| No | 36,134 | 63 | 51,138 | 56 |  |
| Yes | 21,435 | 37 | 40,477 | 44 |  |
| Received Medicaid through Spenddown |  |  |  |  | p<0.001 |
| No | 57,111 | 99 | 89,597 | 98 |  |
| Yes | 458 | 1 | 2,018 | 2 |  |
| Admitted to nursing facility |  |  |  |  | p<0.001 |
| No | 53,074 | 92 | 76,147 | 83 |  |
| Yes | 4,495 | 8 | 15,468 | 17 |  |
| 1. Home & Community Based Services 2. QMB – Qualified Medicare Beneficiary; SLMB – Specified Low-Income Medicare Beneficiary 3. SNAP – Supplemental Nutrition Assistance Program | | | | | |
